# The MOLGENIS toolkit: rapid prototyping of biosoftware at the push of a button

**DOI:** 10.1186/1471-2105-11-S12-S12

**Published:** 2010-12-21

**Authors:** Morris A Swertz, Martijn Dijkstra, Tomasz Adamusiak, Joeri K van der Velde, Alexandros Kanterakis, Erik T Roos, Joris Lops, Gudmundur A Thorisson, Danny Arends, George Byelas, Juha Muilu, Anthony J Brookes, Engbert O de Brock, Ritsert C Jansen, Helen Parkinson

**Affiliations:** 1 Genomics Coordination Center, Groningen Bioinformatics Center, University of Groningen & Dept. of Genetics, University Medical Center Groningen, P.O. Box 30001, 9700 RB Groningen, The Netherlands; 2 EU-GEN2PHEN consortium. http://www.gen2phen.org; 3 EU-CASIMIR consortium. http://www.casimir.ac.uk; 4 EU-PANACEA consortium. http://www.panaceaproject.eu; 5 EU-EUROTRANS cosortium. http://www.euratrans.eu; 6 BBMRI-NL, Postzone S4-P, P.O. Box 9600, 2300 RC Leiden, The Netherlands http://www.bbmri.nl; 7 Netherlands Bioinformatics Centre, Geert Grooteplein 28, 6525 GA Nijmegen, The Netherlands http://www.nbic.nl; 8 European Bioinformatics Institute, Wellcome Trust Genome Campus, Cambridge CB10 1SD, UK; 9 Institute for Molecular Medicine Finland, University of Helsinki, Haartmaninkatu 8, FIN-00290, Helsinki, Finland; 10 Department of Genetics, University of Leicester, University Road, Leicester LE1 7RH, UK; 11 Cluster Information Systems, Faculty of Economics and Business, University of Groningen, P.O. Box 800, 9700 AV Groningen, The Netherlands

## Abstract

**Background:**

There is a huge demand on bioinformaticians to provide their biologists with user friendly and scalable software infrastructures to capture, exchange, and exploit the unprecedented amounts of new *omics data. We here present MOLGENIS, a generic, open source, software toolkit to quickly produce the bespoke MOLecular GENetics Information Systems needed.

**Methods:**

The MOLGENIS toolkit provides bioinformaticians with a simple language to model biological data structures and user interfaces. At the push of a button, MOLGENIS’ generator suite automatically translates these models into a feature-rich, ready-to-use web application including database, user interfaces, exchange formats, and scriptable interfaces. Each generator is a template of SQL, JAVA, R, or HTML code that would require much effort to write by hand. This ‘model-driven’ method ensures reuse of best practices and improves quality because the modeling language and generators are shared between all MOLGENIS applications, so that errors are found quickly and improvements are shared easily by a re-generation. A plug-in mechanism ensures that both the generator suite and generated product can be customized just as much as hand-written software.

**Results:**

In recent years we have successfully evaluated the MOLGENIS toolkit for the rapid prototyping of many types of biomedical applications, including next-generation sequencing, GWAS, QTL, proteomics and biobanking. Writing 500 lines of model XML typically replaces 15,000 lines of hand-written programming code, which allows for quick adaptation if the information system is not yet to the biologist’s satisfaction. Each application generated with MOLGENIS comes with an optimized database back-end, user interfaces for biologists to manage and exploit their data, programming interfaces for bioinformaticians to script analysis tools in R, Java, SOAP, REST/JSON and RDF, a tab-delimited file format to ease upload and exchange of data, and detailed technical documentation. Existing databases can be quickly enhanced with MOLGENIS generated interfaces using the ‘ExtractModel’ procedure.

**Conclusions:**

The MOLGENIS toolkit provides bioinformaticians with a simple model to quickly generate flexible web platforms for all possible genomic, molecular and phenotypic experiments with a richness of interfaces not provided by other tools. All the software and manuals are available free as LGPLv3 open source at http://www.molgenis.org.

## Background

High-throughput technologies have boosted biological and medical research and the need for software infrastructures to manage and process the large datasets produced is widely accepted [1-3]. Bioinformaticians are under continuous pressure to both tackle the complexity and diversity of new biological systems and analytical methods and to translate these quickly into flexible informatics infrastructures, while keeping up with the unpredictable evolution of molecular biotechnologies and the increasing scale of experiments. While standardization of tools and data formats in open source projects like the Generic Model Organism Database, GMOD [4], and the Open Bioinformatics Foundation, OBF [5], have been indispensable in reducing the development efforts needed via reusable and easy to integrate components, new research must also be quickly accommodated, for which efficient software variation mechanisms are needed.

**Figure 1:**
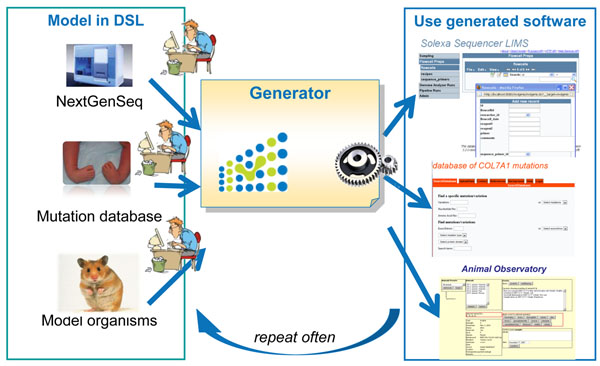
**Model-driven development.** Many minor and major changes have to be written in software code before a ‘standard’ software infrastructure accommodates a particular research. Using ‘model-driven’ development methods a bioinformatician only needs to model what is needed for his experiment using a therefore optimized domain specific language (DSL). Generators quickly produce all the software logic to compose a full software infrastructure that accommodates these needs. When experimental needs change, a bioinformatician can (re)run the same generator with an adapted model file to quickly produce another variant of software infrastructure. This vastly reduces ‘time-to-research’ and enables bioinformaticians to quickly develop a suite of software infrastructures, with each variant accommodating a specific research task, while still on track to reuse, integrate and share the best standard features with other labs and bioinformaticians.

Figure 1 outlines the ‘model-driven’ development method that several bioinformatics projects adopted in recent years to enable fast and flexible infrastructure development [1], for example Taverna and Galaxy for analysis workflows [6,7], CCPN for processing tools [8], and the early MOLGENIS for biological data management [9]. See our review [1] for a more complete overview. This method consists of three components: extensible ‘standard’ software that provides a vast array of reusable components; a high-level modeling language (domain-specific language, DSL) to specify biology-specific customizations to this software; and a software code generator to automatically translate (or execute) such custom models into all lower level program logic of the complete working software, saving all the effort needed to write the software by hand.

In this paper we present the evolution of MOLGENIS into a generic, model-driven toolkit for the rapid generation of bespoke, data-intensive biosoftware applications [10]. We demonstrate step-by-step how bioinformaticians can use a domain-specific language to efficiently model the biological details of their particular biological system, and use MOLGENIS software generation tools to automatically generate a web application tailored to the experiments of their biologists, building on reusable components. Next, we evaluate the results of these methods in the development of a range of MOLGENIS applications [9,11-15], that is, software applications generated using the MOLGENIS toolkit. We found up to 30 times efficiency improvement compared to hand-writing software, while providing a richness of features practically unfeasible to produce by hand but not yet provided by related projects. We conclude by inviting the bioinformatics community to add more MOLGENIS models, components and generators to quickly generate all the software infrastructures biologists want to have.

## Methods

The MOLGENIS toolkit is based on the method of model-driven development which emerged in the 1990s from the computer industry. The key to success is the clear scope of the toolbox (i.e., what family of software applications should be produced with it) and separating which features should be fixed (e.g., reusable components common to all MOLGENIS applications) and which features should be variable (i.e. modeled and generated per MOLGENIS application instance), a process known as domain analysis [16]. Below we discuss MOLGENIS’ initial domain analysis, its modeling language, generators and reusable components.

### Domain analysis

Table 1 summarizes the initial set of features we required from MOLGENIS information systems when we started; it explains why these features are indeed required, and describes what parts of the features are common and variable over experiment databases. To obtain this picture, we analyzed 20 existing microarray databases next to many requirements interviews, see Table 1 in [9].

**Table 1:** Common and variable features of MOLGENIS information systems.

MOLGENIS Features (F)	Common parts (C) and Variable parts (V)
**F1. Data** Store and find lab activities, datasets and biomaterials.	C1. Logic to add, update, remove, find and count data entities in a database; read and write data files. V1. Data structures that suit the research, e.g., samples in a clinical lab have a “tissue” while microbe samples do not.

**F2. Control** Manipulate lab entities such that they suit the research process.	C2. Logic to select, navigate (first, previous, next, last), find (filter), and edit data entities (using the logic of C1). V2. Control structures that suit the research, e.g., experiments are shown with a menu with sub-forms for Samples and Hybridizations.

**F3. View** View entities and control interactively (via the Internet).	C3 Presentation of logic that shows F1 and F2 with usable layout and formatting. V3. Presentation of structure of the specific entity (V1) and control structure (V2) of a system variant via the Internet (option to have this in company style).

**F4. Security** Ensure that the right people get access to the right results.	C4. To manage users, roles and privileges and have authentication and authorization in place. V4. To set Roles and Privileges to entities and controls, e.g., only spotters (role) are allowed to add arrays (privilege).

**F5. Extensibility** Allow addition of components for data processing and visualizations.	C5. To have a plug-in mechanism to integrate external programs so that these programs can benefit from entity and control logic. V5. To extend a system variant with logic beyond the family, e.g., analysis scripts, quantification file validation, complex data aggregation, and export to files.

The second step was to implement the common and variable parts, which we started with a prototype. Here we applied the *don’t repeat yourself* principle (DRY) [17]: every piece of design knowledge must have a single, unambiguous, authoritative representation. We therefore searched through the prototype software code. If we found *identical* pieces then we put them into the library of reusable components. If we found *very similar* pieces of software code, we put the common parts into a generator and the variation points into the modeling language. In each subsequent step we evolved the MOLGENIS generator, only incorporating new functionality when we *repeatedly* needed it.

During the next six years of using the MOLGENIS generator we added numerous functions and optimizations, such as filters for the data, viewing data as a ‘matrix’, downloading data as CSV files, enabling programming interaction via R and web services, and so on. The generators ensure that ‘old’ MOLGENIS application variants can benefit from these improvements: when a MOLGENIS instance is re-generated, these improvements are automatically integrated into the new version.

### Modeling language

**Figure 2:**
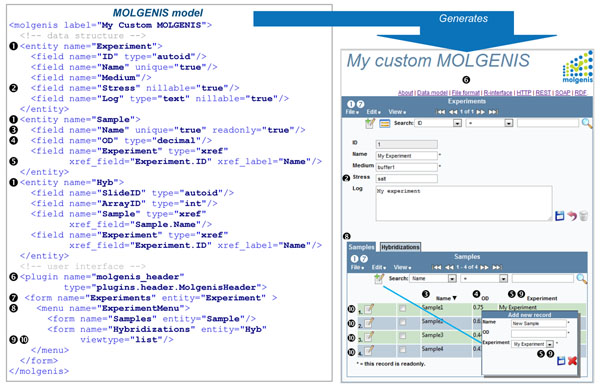
**Example model.** The detailed software needed for an experiment can be described in domain-specific language (DSL, left). The MOLGENIS generator reads the model and automatically produces the custom software infrastructure specified (right). The screenshot includes example data. See main text for a description of the numbers.

Figure 2 shows how a custom MOLGENIS application can be defined in a single file. The file is written in MOLGENIS’ modeling language. This enables compact specification of *what* experiment database is needed, i.e., to declare how an experiment is organized in terms of data types and their relationships and how these data are to be shown on the screen. Figure 2 shows the following features: Three data *entities*: ❶Experiment, Sample and Hybridization; the Experiment entity has six *fields*, including ID, Medium and Stress (because it needs to administrate microbe experiments). To minimize the modeling work we choose sensible defaults in the domain-specific language, a principle known as *convention over configuration*: each field has to be set to a value by the researcher unless specified to be nillable❷; field can be edited (updated) unless specified to be read only❸; each field is default of type ‘string’ (a variable character string of length 255) unless otherwise specified to e.g., ’decimal’❹; and fields can be defined as having a relation to fields in other entities via a cross-reference (xref)❺. The user interface consists of a *plugin*❻ that renders the MOLGENIS header and tool menu; one user interface *form*❼ to control Experiments, with a sub *menu*❽*,* consisting of two *child forms* for Samples and Hybridizations. Child forms are automatically linked to the parent form based on cross references, e.g., the field ‘Experiment’ of ‘Sample’ references to the ‘ID’ of an ‘Experiment’❾. By default, forms show each entity as one-record-per-screen unless specified as a list❿. The modeling language includes advanced object-orientation features like inheritance, as well as extensive help to document your model (not shown).

One can think of MOLGENIS’ modeling language as a ‘domain-specific language’ (DSL) that is optimized to efficiently express a particular problem, task or area [18,19], in this case to compose biosoftware infrastructures. The level of abstraction is raised, so no lengthy, technical or redundant details on *how* each feature should be implemented in general programming languages have to be given [20,21]. Examples of other domain-specific languages include R/Splus for statistics, MatLab for mathematics, SQL for databases, HTML for layouting, and now MOLGENIS’ modeling language for biological software infrastructures.

In most cases, knowledge of the DSL is all that is needed to produce a custom MOLGENIS application variant. The domain-specific language was implemented using XML so that model files can be edited using off-the-shelf XML editors. However, you may want to include hand-programmed components into a particular MOLGENIS instance. For example, for the eXtensible Genotype And Phenotype (XGAP) database application of MOLGENIS [11], we developed a ‘MatrixViewer’ that builds on the generated components, which saved us the work of writing the plug-in from scratch. This requires a model sentence that points to the ‘plug-in’ (allowing it to be seamlessly integrated) as well as hand-programming of the plug-in itself.

### Reusable components

**Figure 3:**
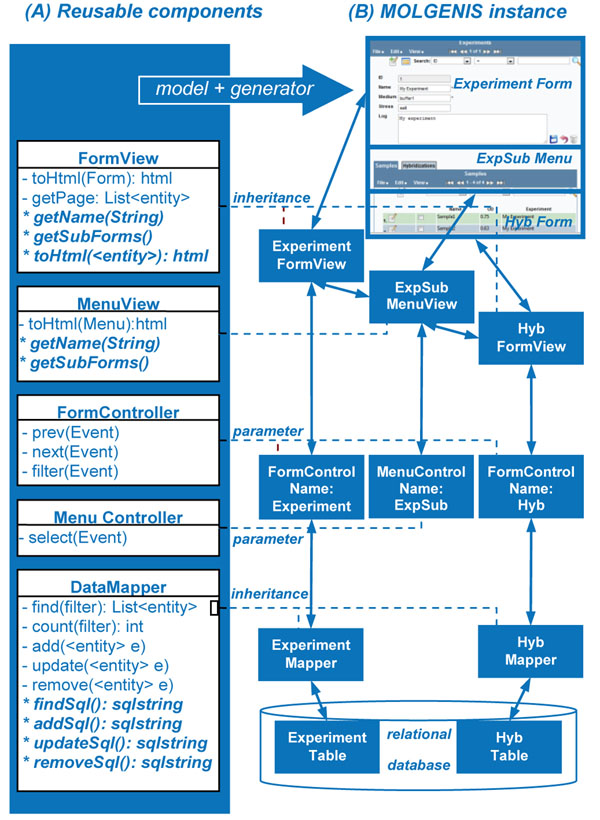
**Reusable components.** (A) shows finished and semi-finished components that provide reusable features for displaying screens (FormView and MenuView), handling user requests (Form- and MenuController), and reading and writing to the database (DataMapper). (B) shows components of a completed software variant as described in Figure 2. Only the ‘differences’ needed to be added using systematic variation mechanisms (dotted lines) such as inheritance or parameterization.

Each MOLGENIS application follows the widely accepted three-layered architecture design of web applications. Figure 3 summarizes some of MOLGENIS’ reusable components and their variation mechanisms. MOLGENIS’ reusable components provide building blocks with a modular structure, which allows them to be assembled in diverse combinations, similar to prefabricated houses that are built from modular walls instead of bricks. Some building blocks are semi-finished and need to be ‘completed’ before use (which is automated in MOLGENIS via the generators and inheritance). We based the design of MOLGENIS on industry-proven design patterns from the ‘patterns for enterprise application architecture’ (PEAA), a catalog of proven solutions for software design problems that we used as a guideline [22]. The logic of the reusable components is implemented using Java (http://java.sun.com); the HTML layout for the user interface is encoded in Freemarker templates (http://freemarker.sourceforge.net/); and the database back-end using MySQL, PostgreSQL or HSQLDB.

### Generators

**Figure 4:**
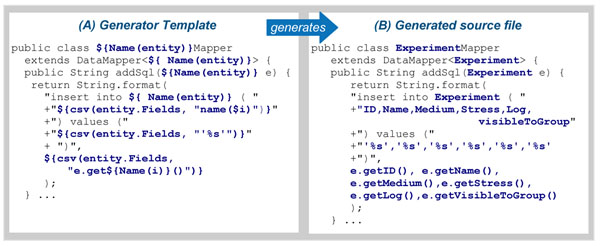
**Example generator.** MOLGENIS generators are implemented as templates. This example shows the generator for a database component (A). This template is applied to each <entity> in the model to generate many complete DataMappers that would otherwise need to be written by hand. (B) shows an example of the generated source files, in this case for <entity name="Experiment"> as described in Figure 1. The command **$Name(entity)** translates to the name of the entity (“Experiment”) and command **${csv($entity.Fields, x)}** means that command ‘**x**’ is applied to each field of the entity and returned as a comma separated string (csv).

The generators are compact specifications of *how* each database feature should be implemented. The MOLGENIS toolkit now has over 20 generators, but normal users will never need to take a look inside. However, for readers wanting to create their own generators, Figure 4 provides an example of the simple, text-based, generators we use. Each generator consists of two files: a Freemarker template that describes the code to be generated (similar to that shown in Figure 4a) and a Java ‘Generator’ class that controls the generating process. A new generator can be developed as follows: first write some examples of the desired programs by hand, where possible using similar patterns (see Figure 4b) and mark which parts are variable between them. Then copy one of these examples into a *generator template* (text file) and replace all variable parts with ‘holes’ that are to be filled by the code generator based on parameters from DSL (see Figure 4a). At each generation, the template is then automatically copied and the ‘holes’ filled, based on parameters described in the domain-specific language, saving much laborious manual work.

## Results

To start generating your own MOLGENIS application, you can download a ready-to-use ‘workspace’ from http://www.molgenis.org, which can be edited using the commonly used Eclipse integrated development environment (IDE) tool (http://www.eclipse.org). Extensive manuals are available to help install the Java, MySQL, Tomcat and Eclipse software needed and to learn how to walk through the Eclipse workspace to edit models and generate and run MOLGENIS instances; most new users can complete this part in about three hours. Below we summarize the output you can expect as well as recent experiences from using this toolbox. Detailed examples on how these features can be used to support actual microarray or genetical genomics experiments can be found in [11,14,15].

### Expected output

**Figure 5:**
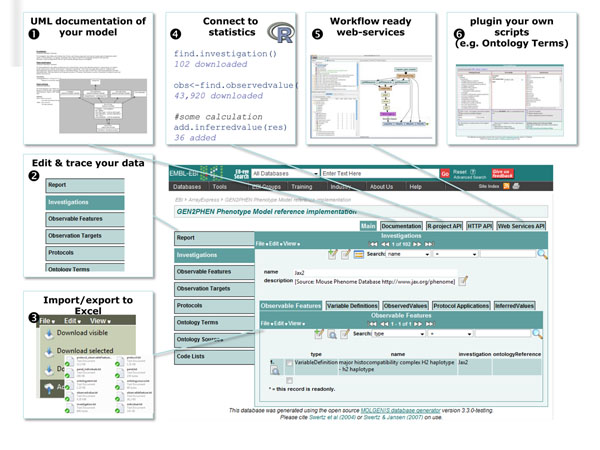
**Expected output.** Overview of a typical MOLGENIS application, in this case customized in EBI style. See main text for a description of the numbers.

After completing a MOLGENIS model and running the generator as described above, you have a ready-to-use software application. Figure 5 summarizes the features you get when running the generated result as a web application: a fully functional system where researchers can upload, manage, browse and query their biological data that conform to the model, optionally enhanced with analysis tools to explore and annotate (depending on the plug-ins).

An important feature is human readable and printable documentation of your model, including a graphical overview showing relationships in UML❶ which is of great use when still designing and discussing the model in a team. The next step is typically using the web user interface to populate and test your application with real data❷. To enable batch loading from a spreadsheet application such as Excel, the system comes with a tab-delimited import/export tool tailored for your data which you can use from the user interface as well as via a command-line tool; i.e., the headers of your Excel file have to match the fields you have defined in the model, ❸. In our experience, most computational biologists greatly appreciate the use of the R interface to load, analyze and re-store data from within the R statistical environment❹ with web services to connect to workflow tools❺. Finally, advanced programmers may want to customize the layout or integrate their own scripts into the user interface, that is, create plug-ins that are seamlessly integrated with the generated software❻. Typical examples here are the integration of R scripts that produce graphical overviews of the data, enabling them to be run by non-technical research colleagues. Alternatively, you can use SOAP, REST and RDF interfaces for integration with workflow tools like Taverna, or for use with commonly used JavaScript frameworks like jQuery to create ‘Web 2.0’ interactive websites. When satisfied with your MOLGENIS system, it can be shared as a simple JAR executable using an embedded web server, or as a WAR file that can be run on public web servers.

### Applications

Since the earliest MOLGENIS application [9], we have successfully evaluated use of the MOLGENIS toolkit to build a wide range of biomedical applications [11-15], ranging from sequencing to proteomics, including:

• XGAP: an eXtensible Genotype And Phenotype platform [11] for systems genetics (GWAS, GWL) to store all kinds of *omics data ranging from genotype to transcript and protein data. XGAP comes with plug-ins to view large data matrices and run processing tools on a cluster. See http://www.xgap.org

• Pheno-OM: to integrate any phenotype data from locus-specific annotations to rich biobank cohort reports with the help of the OntoCAT ontology toolkit to create semantic mappings between related data items [23]. See http://www.ebi.ac.uk/microarray-srv/pheno

• FINDIS: a mutation database for monogenic diseases belonging to the Finnish disease heritage. See http://www.findis.org/molgenis_findis/

• HGVBaseG2P: the data management and curation interface complement for HGVbaseG2P, a central database of genotype to phenotype association studies [12]. See http://www.hgvbaseg2p.org

• MAGETAB-OM: a microarray experiment data platform based on the MAGE-TAB data format standard to create a local microarray repository that is compatible with the public ArrayExpress and GEO repositories. See http://magetab-om.sourceforge.net/

• NordicDB: the database of high-density genome-wide SNP information from 5,000 controls originating from Finnish, Swedish and Danish studies [13]. See http://www.nordicdb.org

• DesignGG: a web tool to optimally design such genetical genomics experiments [14]. See http://gbic.biol.rug.nl/designGG/

More MOLGENIS applications can be found at http://www.molgenis.org. Each of these MOLGENIS projects reported major benefits from the short cycle from model to running system to enable quick evaluation (500 lines of model XML replaces 15,000 lines of programming code) and use of the batch loading of data to evaluate how the newly built system works with real data. More often than not, MOLGENIS helped in finding inconsistencies in existing data that would otherwise have gone unnoticed, leading to experimental errors. In our experience, a typical MOLGENIS generator run gives you about 90% of the application that is desired ‘for free’, with the remaining 10% typically filled in using plug-ins that are written by hand. The MOLGENIS toolkit has also been used to extend or replace existing software applications: the ExtractModel tool allows you to generate a MOLGENIS application from an existing database, which can then be run side-by-side with code developed previously, providing the best of both generated and hand-written worlds.

### Richness of features

MOLGENIS provides a richness of features not yet provided by other projects: BioMart [10,24] and InterMine [25] generate powerful query interfaces for existing data but are not suited for bespoke data management; Omixed [26] generates programmatic interfaces onto databases, including a security layer, but lacks user interfaces; PEDRO/Pierre [27] generates data entry and retrieval user interfaces but lacks programmatic interfaces; and general generators such as AndroMDA [28] and Ruby-on-Rails [29] require much more programming and configuration efforts compared to tools specific to the biological domain. Turnkey [30] seems to come close to MOLGENIS, having GUI and SOAP interfaces but lacks auto-generation of R interfaces and file exchange format.

## Conclusions

In a recent perspective paper [1] we evaluated the general benefits and pitfalls of model-driven development, such as the ability to develop infrastructure in short cycles to get the application right, ensuring developers and biologists are thinking along the same lines and increasing quality and functionality for all. We further evaluated applying this method to both microarray and genetical genomics experiments [9], [11].

Here we have presented MOLGENIS in detail and reported the results of using this method against a wider range of applications. We conclude that using model-driven methods enables bioinformaticians to build biological software infrastructures faster than before, with the additional benefit of much easier sharing of models, data and components. Much less time is spent on customizing and gluing together individual components. The result is of higher quality because fewer incidental errors creep into the applications as a consequence of the automated procedures; best practices are applied instead of reinvented. And you do not need heavy-weight technology to implement a model-driven generator: simple text-based templates suffice to create biological software generators.

As a next step we want to expand the MOLGENIS toolkit to also generate data processing tools, including user friendly interaction, building on other ‘model-driven bioinformatics’ projects in this area, such as Taverna [6] to model/execute analysis workflows and Galaxy [7] to generate user interfaces for processing tools. We hope that many bioinformaticians will enforce our open source efforts and share their best models, plug-ins and generators at http://www.molgenis.org, so that, in time, every biologist may find a MOLGENIS variant that suits his/her needs.

## Availability and requirements

Project name: MOLGENIS

Project homepage: http://www.molgenis.org

Operating systems: Windows, Linux, Apple

Programming language: Java JRE 1.5 or higher

Other requirements: MySQL or Postgresql, Tomcat or other J2EE container

License: GNU Lesses General Public License version 3 (GNU LGPLv3)

Any restrictions to use by non-academics: No

## Abbreviations

API: application programming interface; CPNN: collaborative computing project for NMR; CSV: comma separated values; DesignGG: experimental design of genetical genomics software; DRY: principle of don’t repeat yourself; DSL: domain specific language; EBI: European Bioinformatics Institute; FINDIS: finish disease database; GEN2PHEN: EU project to unify human and model organism genetic variation databases; GMOD: generic model organism database project; GUI: graphical user interface; GWAS: genome wide association study; GWL: genome wide linkage analysis; HGVBaseG2P: human genome variation database of genotype-to-phenotype information; HTML: hypertext markup language; IDE: integrated development environment; JAR: Java Software Archive; LGPL: lesser general public license; MAGE-TAB: microarray gene expression tab delimited file format; MOLGENIS: molecular genetics information systems toolkit; NordicDB: Nordic Control Cohort Database with harmonized SNP information from Denmark, Estonia, Finland and Sweden; OBF: Open Bioinformatics Foundation; OntoCAT: ontology common API toolkit; PEAA: patterns for enterprise application architecture; QTL: quantitative trait locus; RDF: resource description format; REST: representative state transfer web services; SNP: single nucleotide polymorphism; SOAP: simple object access protocol; SQL: structured query language; UML: uniform data modeling language; WAR: web application archive file; XML: extensible markup language; XGAP: extensible genotype and phenotype software platform.

## Competing interests

The authors declare that they have no competing interests.

## Authors' contributions

MAS and EOB conceived the method and designed and implemented the first MOLGENIS generator suite. MAS, MD, KJV, TER, AK, JL, DA, GB, GAT, JM, and TA participated in the development of the MOLGENIS toolkit and/or have been developing applications using MOLGENIS as a platform. RCJ, HP, GAT, and AJB have been feeding requirements to steer future development. MAS drafted the manuscript. All authors read and approved the final manuscript.
